# Effect of Vocal Nerve Section on Song and ZENK Protein Expression in Area X in Adult Male Zebra Finches

**DOI:** 10.1155/2012/902510

**Published:** 2012-11-26

**Authors:** Congshu Liao, Dongfeng Li

**Affiliations:** Key Laboratory of Ecology and Environmental Science in Higher Education of Guangdong Province, School of Life Science, South China Normal University, Guangdong, Guangzhou 510631, China

## Abstract

ZENK expression in vocal nuclei is associated with singing behavior. Area X is an important nucleus for learning and stabilizing birdsong. ZENK expression is higher in Area X compared to that in other vocal nuclei when birds are singing. To reveal the relationship between the ZENK expression in Area X and song crystallization, immunohistochemistry was used to detect ZENK protein expression in Area X after the unilateral vocal nerve (tracheosyringeal nerve) section in adult male zebra finches. Sham operations had no effect on song. In contrast, section of unilateral vocal nerve could induce song decrystallization at the 7th day after the surgery. The spectral and the temporal features of birdsong were distorted more significantly in the right-side vocal nerve section than in the left-side vocal nerve section. In addition, after surgery, ZENK expression was higher in the right-side of Area X than in the left-side. These results indicate that the vocal nerve innervations probably are right-side dominant. ZENK expression in both sides of Area X decreased, as compared to control group after surgery, which suggests that the ZENK expression in Area X is related to birdsong crystallization, and that there is cooperation between the Area X in AFP and syrinx nerve.

## 1. Introduction

The anterior forebrain pathway (AFP) begins with the projection from promoter nucleus high vocal center (HVC) to Area X and is essential for song learning but not for song production [1, 2]. Area X is the first relay of the AFP, strongly similar to mammalian basal ganglia [3, 4]. In songbirds, Area X in the medial striatum (Mst), which plays an important role in promoting stabilization of temporal structure of song and sequence of syllables, is responsible for long-term potentiation (LTP) during either adult or juvenile period [5, 6]. 

The immediate-early gene *zenk* encodes a zinc-finger transcription factor that is involved in sound evoking. *zenk* can promptly respond to calls or songs of songbirds [7, 8]. Induced expression of ZENK by singing can be detected in nuclei of HVC, arcopallium (RA), lateral magnocellular nucleus of the anterior nidopallium (lMAN), dorsomedial nucleus of the intercollicular complex (DM), and Area X, with the highest level in Area X and the lowest in RA and lMAN [9–13]. Additionally, the amount of ZENK expression in song nuclei was proportional to the number of syllables in singing per time unit [13–15]. 

Similar to human, songbirds learn vocalization through imitation, starting at an early stage from birth to 25 d, which is called “subsong.” When the voice imitation becomes recognizable, the songbirds enter “plastic song” stage (25 ~ 90d). Finally, when the imitations are perfected and songs become less variable, the stage is called “stable song" [2]. Adult male zebra finches produce highly stable songs via auditory feedback. Previous evidence indicates that zebra finch has right side syrinx control sound dominance and section of the right side of syrinx nerve, which following perturbs auditory feedback would disrupt this stability of song pattern, a process known as decrystallization [16, 17]. However, whether or not the ZENK expression in Area X is related to the song decrystallization remains unknown. In this paper, we revealed the relationship between the ZENK expression in Area X and song crystallization by examining the ZENK expression in Area X after unilateral vocal nerve section. The results will help us better understand the functional relationship between forebrain and lower brain. 

## 2. Materials and Methods

### 2.1. Materials

#### 2.1.1. Animals and Experimental Groups

Experiments were performed on a total of 14 adult (>90 days old) male zebra finches (*Taeniopygia guttata*). All experiments were carried out in accordance with the university and national animal guidelines. Zebra finches were obtained from a local breeder. Birds were housed in stainless steel cages (23.5 × 22.5 × 27.5 cm); each of the cages contained a pair of male and female birds. The birds were divided into four main experimental groups: “right side of vocal nerve section 7d” (*n* = 4 birds); “left side of vocal nerve section 7d” (*n* = 4 birds); “sham-operated group” (*n* = 3 birds); control group (*n* = 3 birds). 

### 2.2. Methods

#### 2.2.1. Tracheosyringeal Nerve Section

Before vocal nerve section, songs of all birds were recorded in the presence of adult female birds. Birds were then anesthetized with 10% chloral hydrate (0.02 mL/10 g), and a 3–5 mm section of the right (or left) tracheosyringeal nerve was removed, followed by a restoring of the skin. Postmortem visual inspection in random bird samples showed that the nerve could not recover to normal during the following several weeks, even months. After surgery, individual bird was reared in a solitary cage for 3 days and kept isolation from others so that they could not hear and see each other. The sham operations underwent the same surgery but without section of the vocal nerve. 

#### 2.2.2. Song Recording

The songs of adult male zebra finches were recorded for 14 ± 3 days before the experiment. Male birds within a test room were completely isolated acoustically, but visually, from the female birds from window in a neighboring room. The songs were recorded between 8:30 AM and 16:00 PM. In last test day, after birds stopped singing for 30 min, each male was decapitated. Songs were recorded before and at 3, 7, 14, and 21d after vocal nerve section in the presence of adult female zebra finch. All songs analyses described here were performed in the presence of adult female only. Song recording and editing were performed using custom software (Cool Edit 2000). 

#### 2.2.3. Spectral and Temporal Analyses of Songs

Sonogram and waveform were subsequently created using the software wavesurfer 2.0 (Hamming window; FFT size: 8192). To quantify distortion in spectral and temporal features of song after vocal nerve section, relative amplitude (RA), principal frequency (PF), sound duration (SD), and interval of single syllable (ISS) of the songs were used to measure the differences between before and after surgery [18]. 

#### 2.2.4. Histology and Immunohistochemistry

Following the 30-min silence period, birds were given an overdose of 10% chloralic hydras and perfuse with 0.1 M phosphate buffered saline (PBS) followed by 4% par formaldehyde in PBS. Brains were removed, and the gender of each bird was confirmed by inspecting its gonad(s). Following a 1 h postfix in paraformaldehyde, the brains were treated for overnight in 30% sucrose in PBS at 4°C. Alternate series of 30 *μ*m coronal sections were collected and stored in cryoprotectant for overnight at 4°C then at −20°C until Nissl stain or immunohistochemical processing.

Endogenous peroxidase was blocked by incubation in 0.3% H_2_O_2_ in PBS. Immunohistochemistry for ZENK was performed by using ZENK antibody (1 : 2000, Santa Cruz Biotech; catalog #sc-189; 0.1 g/mL) for overnight at 4°C. Following primary antibody incubation, a goat anti-rabbit secondary antibody, labeled with either DAPI or FITC (Sigma), was added. The protein was visualized and photographed under a Leica DMI 3000B microscopy.

#### 2.2.5. Quantification of ZENK Expression

Quantification of ZENK expression was performed as described previously [10]. Briefly, 1-2 particular parasagittal planes were randomly chosen, and ZENK-labeled nuclei within Area X were counted for all birds. Area X is very clearly defined as an approximately comma structure distinct from the MSt (medial striatum) on both sides (as depicted by the diagram in Figure 3(a)). All ZENK-labeled nuclei present within Area X in each section were counted, and the resulting number was the total Area X in that particular section to generate a density value, and the results were averaged. 

#### 2.2.6. Statistical Analysis

Paired two-sample *t*-test (Origin 8.0) was used to statistically determine significance of the differences between before and after nerve section. Left and right nerve section data were analyzed separately. ANOVA analysis was performed to compare the variance of the effect of ZENK expression in the left and right in Area X before and after surgery. Changes are considered significant when *P* < 0.05 and very significant when *P* < 0.01. Data are presented as mean ± SD.

## 3. Results

### 3.1. Spectral and Temporal Features of the Birdsongs before and after Unilateral Vocal Nerve Section

The songs of the tested birds were recorded before and after the vocal nerve section for several weeks, and their spectral and temporal characteristics of songs were compared. 

**Figure 1:**
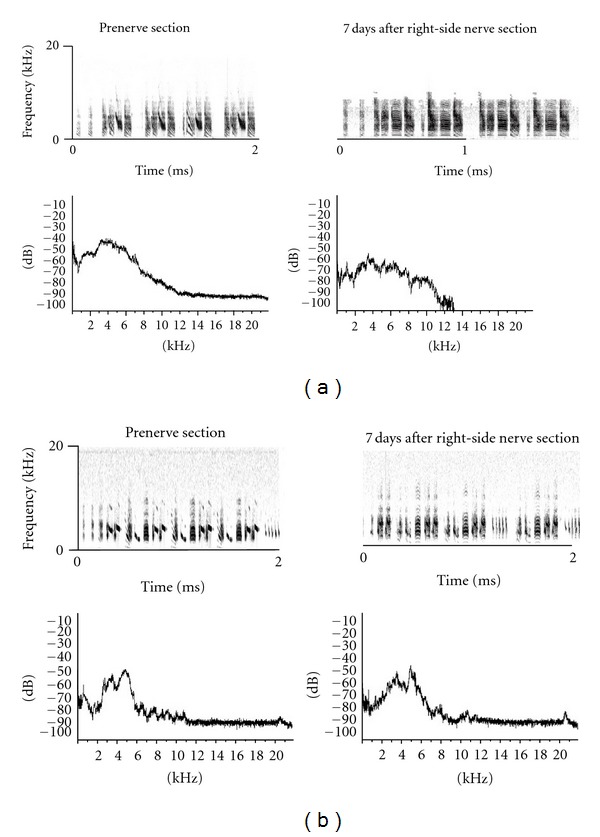
Unilateral section of vocal nerve leading to distortion of the structure of song. (a) The sonogram (top) and spectrum (bottom) before and after right-side vocal nerve section. (b) The sonogram (top) and spectrum (bottom) before and after left-side vocal nerve section.

Birdsong usually contains at least three motifs. Each syllable in a motif contains its duplicates from high frequency to low frequency as harmonics. The results indicated that the songs of the control group contained high- and low-frequency components. RA varied significantly in low frequency but stabilized in high frequency. High-frequency components were distorted after right-side vocal nerve section. RA was lost after 13 kHz, and low-frequency components were more variable than those before surgery in spectrum (Figure 1(a)). There was no distortion in harmonics after left-side vocal nerve section, but the syllable sequences changed by missing and increment in sonogram. The components of birdsong were distorted mainly in the range of 2 ~ 4 kHz with increase in low-frequency components (Figure 1(b)).

**Figure 2:**
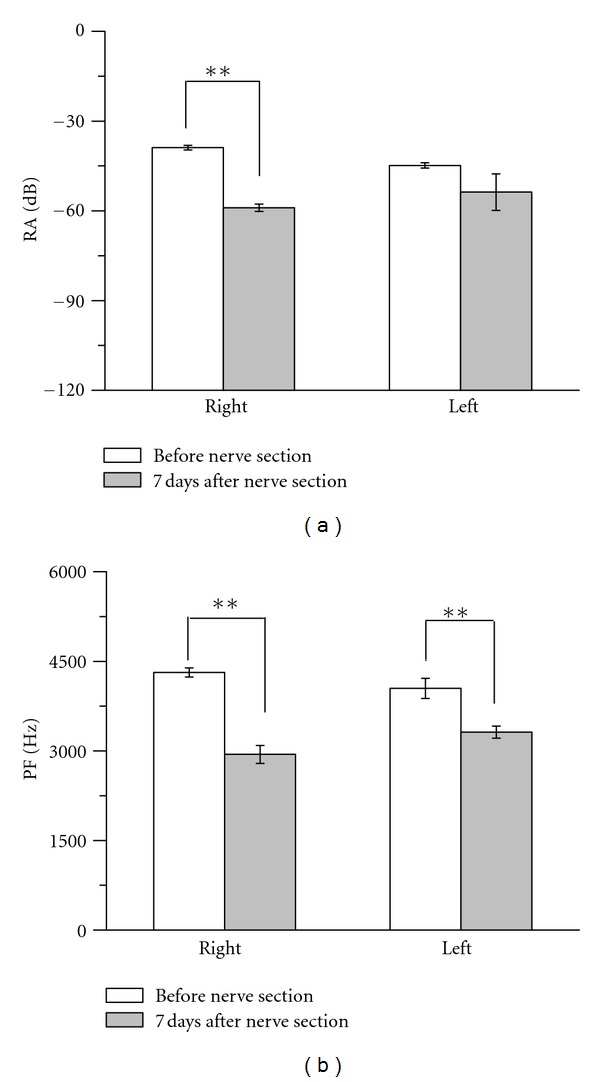
Changes in RA and PF of birdsong before and after right- or left-side vocal nerve section. (a) RA comparison before and after one side of vocal nerve section, before and after right-side vocal nerve section (*P* < 0.01), before and after left-side vocal nerve section (*P* = 0.155). (b) PF comparison before and after one side of vocal nerves section, before and after right-side vocal nerve section (*P* < 0.01), before and after left-side vocal nerve section (*P* < 0.01).

The analysis of the spectral characteristic before and after right-side vocal nerve section indicated that RA decreased from −38.89 ± 3.55 dB to −59 ± 5.46 dB (*P* < 0.01, *n* = 20/songs) (Figure 2(a)), and PF decreased from 4314.65 ± 347.019 Hz to 2941.9 ± 672.02 Hz (*P* < 0.01) (Figure 2(b)). Both of these decreases were significantly at *P* < 0.01 level. However, the analysis on the left vocal nerve section, only PF was significantly decreased after the surgery from 4046.6 ± 755.83 Hz to 3312.35 ± 450.03 (*P* < 0.01, *n* = 20/songs) (Figure 2(a)), whereas the reduction in RA from −44.83 ± 4.05 to −53.77 ± 27.28 (*P* = 0.155), was not significant (Figure 2(b)).

The ranges of SD and ISS for the control birdsongs were 47.7 ± 5.83 ~ 110.25 ± 20.36 ms and 0 ~ 41.02 ± 12.57 ms, respectively. After right-side vocal nerve section, SD_min⁡_, ISS_min⁡_, and ISS_max⁡_ had an increasing trend, but SD_max⁡_ had a trend of decrease, suggesting that single syllable of birdsong lasted longer and the intervals between syllables become longer than before the section. On the other hand, after left-side vocal nerve section, SD_min⁡_ increased, whereas SD_max⁡_ decreased. The ranges of ISS_min⁡_ and ISS_max⁡_ were expanded. Either side of vocal nerve section showed similar trend in temporal features (Table 1). 

### 3.2. ZENK Expression in Area X before and after Vocal Nerve Section

**Figure 3:**
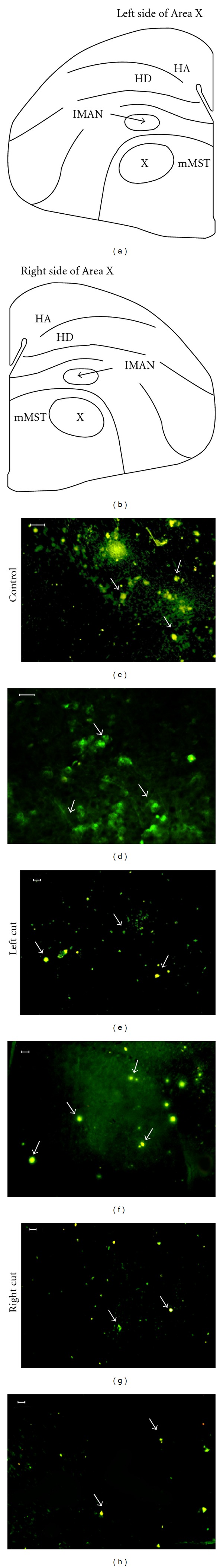
The distribution of ZENK labeled in Area X. ((a), (b)) Area X is a large nucleus distinct from the surrounding Mst. Area X is detectable in the right- and left-side brain of the male zebra finch. ((c), (d)) Before nerve section both sides of the ZENK expression in Area X; ((e), (f)) ZENK expression in Area X at the 7th day after left vocal nerve section; ((g), (h)) ZENK expression in Area X after right vocal nerve section at the 7th day. The small arrow indicates the ZENK-positive cells present in Area X (bar: 100 *μ*m).

To examine ZENK expression in the Area X, immunohistochemistry was performed before and after vocal nerve section. The result showed that ZENK immunoreactive neurons in Area X were higher in the surrounding portion than in the medial portion. No difference in the ZENK expression was found between right and left sides of the Area X in the control group (Figures 3(c) and 3(d)). The ZENK expression in Area X in left hemisphere was localized on the bottom portion, whereas in right hemisphere the expression was localized on the top portion of the Area X. ZENK was expressed at the medial and lateral portion of Area X after left-side nerve section, and higher expression of the protein was found on right side than on left side of the Area X (Figures 3(e) and 3(f)). However, the ZENK expression was decreased after right-side vocal nerve section (Figures 3(g) and 3(h)). 

**Figure 4:**
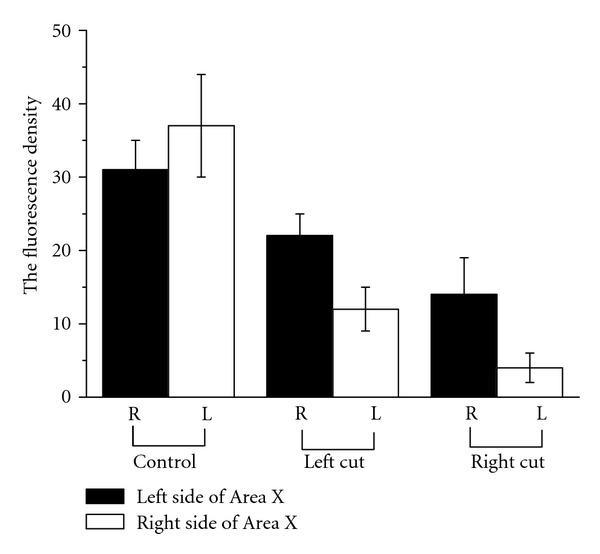
The density ZENK expression in the brains of adult male zebra finches. In control group, there is no difference in both hemispheres. There is right-side dominance of the ZENK expression in Area X after vocal nerve section (bars represent the SD).

Quantitative and ANOVA analyses of the protein expression in term of fluorescence intensity were performed to determine significance of the differences in ZENK expression between the left and right sides of the Area X, before and after surgery on the right-side and left-side vocal nerves. The ZENK expression in both sides of the Area X after surgery, either on the right-side vocal nerve section or left-side vocal nerve section, was significantly lower (*P* < 0.05) than those in the control group (Figure 4). For the control (without nerve section), the higher expression of the protein was found in the left side than in the right-side of the Area X, although the difference was not significant. However, for the right-side vocal nerve section, the right side of the Area X had a significantly (*P* < 0.05) higher expression of the protein than the left side after the surgery. Similarly, for the left-side vocal nerve section, the right-side of the Area X also had a significantly (*P* < 0.05) higher level of the protein expression than the left side after the surgery (Figure 4). These results suggest that after either right-side or left-side vocal nerve section, the protein expression became Area X right-side dominant, as compared to the control (without nerve section), in which the left side had a higher level of the protein expression than the right side. 

## 4. Discussion

This study found that adult male zebra finches could still sing after either the left or right-side vocal nerve was sectioned, suggesting that either side of the vocal nerve alone could control singing by controlling singing muscle [19, 20].

In contrast to the results of Roy and Mooney [16], our results of the effects of unilateral section of vocal nerve section on the definition of decrystallization indicated that vocal nerve section on either side could induce decrystallization of adult zebra finch song starting at the 7th day after surgery. Furthermore, the right-side vocal nerve had more significant effect than the left-side nerve on the birdsong (Figure 1). These results taken suggest that vocal nerves have right-side sound control dominance in adult male zebra finch.

In early 1990s, Mello et al. reported that the ZENK expression in birds' brain is related to long-term potentiation [11]. The Area X is a site where learning song and self-singing song information are gathered and integrated [3, 21]. Singing can induce up to a 60-fold increase in ZENK expression [15]. In this study, we examined the ZENK expression in Area X in zebra finches and found that this gene was expressed in a moderate level on the both sides of the Area X before vocal nerve section. However, after unilateral section of the syrinx nerve either on the right side or the left side, the ZENK expression in both sides of Area X significantly decreased as compared to the control. There are two possible explanations for this change in the ZENK expression. First, the ZENK expression is proportional to numbers of syllables of birdsong within unit time. Because the syllable numbers decrease after the surgery, the expression of the protein decreases. In addition, type of song presentation (either direct or indirect) affects the expression of the *zenk* gene in nuclei [22]. Spectrum analysis shows that undirected songs have a longer prelude and motif and faster release speed of basic syllable than directed songs [23, 24]. We predict that the more songs are stabilized, the less the ZENK protein is expressed and the ZENK expression in adult male zebra finch is related to crystallization of birdsong because the decrystallization of birdsongs after surgery was accompanied by reduction of the ZENK expression. Second, singing behavior of the birds was reduced after surgery, which may also indirectly suppress the expression of ZENK. 

Lateralization of songbird has been investigated for more than a century. Nottebohm [25] found for the first time the asymmetrical control of songs in passerine bird. Since then, substantial reports have suggested that right hemispheres of adult zebra finch have a more significant predominance than left hemispheres, the neural mechanism involved HVC, and the caudal medial nidopallium (NCM) [26, 27]. In contrast, Moorman et al. [28] found that juvenile male zebra finch showed left-side dominance for tutor song but not for unfamiliar song in HVC [28]. It is possible that lateralization is dependent on not only the song learning phase but also the social contexts. Our data show that the control group had higher protein expression in Area X without lateralization. However, the ZENK expression has right-side dominance in Area X after surgery. And also suggest that the opportunity to further study on lateralization of the brain can be performed on molecular and gene levels. The expression of ZENK protein in Area X is relevant to crystallization of spectral and temporal characteristics of the birdsong in male zebra finch, suggesting cooperation between the Area X in AFP and syrinx nerves.

## Figures and Tables

**Table 1:** Measurement of SD_min_/SD_max_ and ISS_min_/ISS_max_ before (pre-) and after (post-) right- and left-side vocal nerve section.

Parameters	Right		Left	
	Pre-R	Post-R	Pre-L	Post-L
SD_min_	47.7 ± 5.83	88.4 ± 12.49	52.96 ± 11.89	66.48 ± 22.7
SD_max_	110.25 ± 20.36	87.15 ± 15.45	108.11 ± 26.03	105.96 ± 47.34
ISS_min_	0	12.15 ± 5.7	0	15.89 ± 3.27
ISS_max_	41.8 ± 16.12	112.65 ± 34.14	44.02 ± 12.57	115.02 ± 23.06

Each measurement is presented as mean ± SD (ms). Pre, control; SD_min_/SD_max_ (ms) minimum/maximum duration of syllable; ISS_min_/ISS_max_ (ms) minimum/maximum interval between syllables.
